# Senescence-associated signature based on immunotherapy response sequencing reveals PPIL3 as target for bladder cancer treatment and prognosis prediction

**DOI:** 10.3389/fimmu.2025.1613056

**Published:** 2025-06-26

**Authors:** Kaixuan Du, Ning Kang, Yuda Lin, Kaipeng Jia, Chong Shen, Zhouliang Wu, Hailong Hu

**Affiliations:** ^1^ Department of Urology, The Second Hospital of Tianjin Medical University, Tianjin, China; ^2^ Tianjin Key Laboratory of Urology, Tianjin Institute of Urology, The Second Hospital of Tianjin Medical University, Tianjin, China

**Keywords:** senescence, immunotherapy, bladder cancer, PPIL3, therapeutic target

## Abstract

**Background:**

Bladder cancer (Bca) remains a major genitourinary malignancy with unmet needs in immunotherapy optimization. Despite advancements in immune checkpoint inhibitors (ICIs), challenges persist, including low response rates and drug resistance. Emerging evidence links tumor cell senescence to immunotherapy efficacy, yet predictive biomarkers are lacking.

**Methods:**

We integrated genomic sequencing of real-world Bca patients receiving low-dose paclitaxel combined with immunotherapy to identify differentially expressed genes (DEGs) between responders and non-responders. By intersecting DEGs with senescence-related gene sets (SRGs), we derived senescence-related DEGs (SRDEGs) and constructed a senescence-immunotherapy model (SIM) via TCGA-based multi-regression analysis.

**Results:**

The SIM, validated across three independent cohorts, demonstrated superior prognostic accuracy for overall survival (OS) compared to clinical parameters. High SIM scores correlated with immunosuppressive tumor microenvironments (TME). Drug sensitivity analysis revealed differential responses to cisplatin and paclitaxel between SIM subgroups. Critically, real-world validation confirmed SIM’s predictive power for immunotherapy response. Multi-omics profiling further highlighted PPIL3 as a hub gene driving senescence and suppressing proliferation. *In vitro* experiments showed elevated expression of PPIL3 facilitated the concentration of senescence markers (SA-β-gal) and inhabited tumor cell proliferation.

**Conclusions:**

This study establishes SIM as a dual-purpose tool for survival prediction and immunotherapy stratification, and suggested that PPIL3 could be a therapeutic target to enhance the efficacy of Bca by regulating senescence.

## Introduction

Globally, Bca is ranked as the ninth most common malignancy overall, the sixth most prevalent among men, the tenth among women (1, 2), and the number of cases was predicted to increase significantly worldwide between 2022 and 2046 (3). Even with the availability of multiple treatment modalities for Bca, the OS for patients remains suboptimal. In the case of non-muscle invasive bladder cancer (NMIBC), standard therapeutic interventions include transurethral resection of bladder tumors (TURBT) and localized perfusion therapies like Bacillus Calmette-Guérin (BCG) immunotherapy. Although BCG therapy demonstrates efficacy in numerous instances, approximately 30-40% of patients exhibit resistance, and relapse rates can reach up to 50% (4, 5). For muscle-invasive bladder cancer (MIBC), the conventional treatment approach typically involves radical cystectomy (RC); however, this procedure is associated with considerable morbidity and a diminished quality of life. Consequently, bladder-sparing treatment modalities have garnered substantial attention over the past few years (6, 7). The advent of immunotherapy and antibody-drug conjugate (ADC) have brought about the adoption of innovative treatment approaches, such as PD-1/PD-L1 inhibitors and RC48-ADC (8), in the management of Bca (9). Previous research indicated that the integration of immunotherapy with chemotherapy has the potential to enhance therapeutic outcomes, particularly in patients with advanced-stage Bca (10). Despite the considerable advancements achieved in the application of immunotherapy for Bca treatment, several limitations persist. Key challenges include suboptimal response rates (11), the lack of fully reliable biomarkers (12), the occurrence of immune-related adverse effects, and the appearance of resistance to medications (13). Consequently, there is an imperative necessity to investigate advanced immunotherapeutic strategies and integrative treatment modalities to establish a promising approach for the comprehensive management of Bca.

The cellular context in which a tumor develops is known as the tumor microenvironment (TME) (14). Previous research indicated that alterations in the TME can significantly influence tumor progression (15), invasiveness, and treatment resistance (16). For instance, tumor-associated macrophages (TAMs) are integral elements of the TME, as they facilitate tumor angiogenesis and suppress anti-tumor immunity, thereby diminishing therapeutic efficacy (17). With the advancement of immunotherapy, strategies aimed at restructuring the TME have increasingly garnered scholarly interest. Consequently, research on the TME is intensifying, and a comprehensive understanding of its composition and function is vital for creating innovative therapies. Addressing the TME may greatly boost the efficacy of cancer treatments and lower the chances of developing resistance (18–20).

Cellular senescence represents a multifaceted biological phenomenon typically induced by cellular stress, damage, or proliferative stimuli. Senescent cells are integral to normal physiological processes and exert significant influence on tumorigenesis and the modulation of the immune microenvironment (21). Previous research indicated that the stable interruption of the cell cycle and the linked secretory function, described as the senescence-associated secretory phenotype (SASP), have the capacity to remodel the tumor-immune microenvironment, consequently affecting antitumor immunity (22). Senescent tumor cells exert multifaceted influences on the TME. They have the capacity to activate anti-tumor immune cells, thereby fostering an anti-tumor immune microenvironment, while simultaneously activating immunosuppressive cells, which play a role in developing an immunosuppressive microenvironment (15). Consequently, a comprehensive investigation into the composition and mechanisms of the tumor microenvironment, as influenced by tumor cell senescence induced by various factors, establishes a foundational basis for the reconfiguration of the tumor microenvironment associated with the SASP. Simultaneously, this exploration significantly contributes to the advancement of multi-faceted research in both enhanced and combined immunotherapeutic strategies.

This study utilizes genome sequencing to investigate Bca patients who have been treated with low-dose paclitaxel in conjunction with immunotherapy in a real-world setting. By exploring the genomic differences between those who respond to immunotherapy and those who do not, we identified DEGs. We jointly analyzed these DEGs with SRGs to obtain SRDEGs. We then developed a predictive SIM based on five genes using data from the TCGA database by applying univariate regression, LASSO regression, and multivariate regression analysis. Subsequently, patients were sorted into high and low categories using the median value of the senescence-related and immunotherapy-related model scores (SIMS), to facilitate an in-depth investigation of the variations in the TME and immunotherapy response between these subgroups. Ultimately, we confirmed the impact of the model risk score on immunotherapy response using independent databases and real-world data. More importantly, we explored the important role of PPIL3 in inducing senescence and inhibiting proliferation of Bca cells.

In conclusion, we aim to develop a senescence-associated signature to enhance the prediction of treatment outcomes and prognosis for patients with Bca and reveal a potential therapeutic target for Bca by regulating cell senescence-related pathways.

## Materials and methods

### Data acquisition

The TCGA datasets were obtained from UCSC Xena, while the GEO datasets were sourced from the National Center for Biotechnology Information (NCBI). Senescence -related datasets were retrieved from the Molecular Signatures Database (MSigDB), and the Imvigor210 datasets were acquired through the R package “IMvigor210CoreBiologies.”

### Patient information

Bca tissue specimens were gathered from patients receiving immunotherapy at the Second Hospital of Tianjin Medical University. Corresponding clinical data were also collected at this institution. The Ethics Committee of the hospital reviewed and approved every study.

### Transcriptome sequencing

To begin, retrieve the Bca tissue sample from storage, either at -80°C or in liquid nitrogen, and proceed with the extraction of total RNA from the sample. The extracted RNA’s concentration and purity are determined through the NanoDrop spectrophotometric method. Each sample is measured two to three times to ensure accuracy, with the average optical density (OD) ratio at 260/280 nm maintained between 1.8 and 2.1, thereby meeting the required standards. The concentration and purity metrics for each RNA sample are meticulously recorded. Following this, the RNA is converted into complementary DNA (cDNA). The cDNA synthesized through reverse transcription is subsequently amplified to yield a sufficient quantity of DNA, which is essential for the construction of a sequencing library. The cDNA is then fragmented and ligated with adapters at both ends. Following this, the library is further amplified using polymerase chain reaction (PCR) to increase its concentration, ultimately resulting in the formation of a cDNA library prepared for sequencing. This library is then analyzed using high-throughput sequencing platforms, enabling the production of comprehensive sequence data.

### Cell culture and transfection

The Bca cell lines of human were obtained from the American Center for Type Culture Collection. Cells were cultured to approximately 70% confluence and then transfected with HitransG P viral infection reagent according to the manufacturer’s instructions.

### Quantitative real-time PCR

The experimental steps for extracting RNA and obtaining cDNA were as described above and in previous studies (23). The primer sequences used in the experiment are shown in the **Supplementary Table 1**.

### Western blot

According to previous experimental methods (23), proteins from Bca cells were extracted, separated and quantitatively analyzed.

### Cell counting Kit-8 assay

According to previous experimental methods (23), using the CCK8 assay to assess the proliferation activity of Bca cell lines.

### Colony formation assay

About 1000 cells were placed in a 6-well plate. After culturing for about 10 days, they were fixed with paraformaldehyde for 15 minutes. More importantly, they were stained with 0.1% crystal violet for 30 minutes. After drying, the number of colonies was counted.

### EdU assay

According to previous experimental methods (23), cell proliferation was assessed according to the EdU detection kit instructions. After incubation with EdU reagent for 2 hours, cells were fixed with paraformaldehyde and permeabilized with Triton X-100. Cells were then treated with reaction mixture for 30 minutes. Finally, cell nuclei were stained with DIPA, observed under a fluorescence microscope, and photographed and counted.

### Immunohistochemistry

The experimental procedures were as described in previous studies (23, 24), and the information of antibodies used in the experiments was shown in the **Supplementary Table 1**.

### Differential gene analysis

This study utilizes genome sequencing to investigate Bca patients who have been treated with low-dose paclitaxel in conjunction with immunotherapy in a real-world setting. A comprehensive bioinformatics analysis was conducted on extensive sequence data acquired through sequencing. Depending on their clinical response to immunotherapy, two groups were formed from Bca patients: responders and non-responders. The analysis of DEGs expression performed in the R programming environment, specifically employing the “DESeq” package, to identify gene sets exhibiting significant variations between the two groups (P<0.05) (**Supplementary Table 2**).

### Unsupervised cluster analysis

Unsupervised cluster analysis was employed to categorize gene expression levels, which was subsequently followed by a comparative evaluation of clinical characteristics across the delineated subtypes. In this study, the R package “ConsensusClusterPlus” (version 1.64.0) was utilized in the clustering purposes, with references offering further methodological context (25, 26). The PAC algorithm identified an optimal K value of 3. Following this, a comparative analysis of the clinical data across the three delineated subgroups was performed, resulting in the conclusion that the gene set is significantly correlated with the OS rate of patients.

### Lasso regression analysis

Lasso regression is a commonly utilized method of regression analysis within the field of statistics (27). The primary objective is to compress coefficients to facilitate variable selection and complexity adjustment, ultimately boosting the predictive accuracy and explanatory power of the model. The R package “glmnet (4.1-8)” (27) was used to perform lasso regression on the selected gene sets (28).

### Cox regression analysis

Cox univariate analysis is a technique employed to assess the influence of a single variable on patient survival or recurrence risk. In this study, we utilized univariate regression analysis to identify each gene within the DEGs that was associated with patient survival, serving as a basis for subsequent modeling. Concurrently, we applied Cox multivariate analysis to evaluate the impact of multiple genes, identified through lasso regression, on patient survival, thereby constructing the final predictive model.

### Survival analysis

Kaplan-Meier survival analysis, a nonparametric technique for estimating survival probabilities from observed survival time data, was carried out with the R package “survival” (version 3.5-5). Additionally, the function of SIM was corroborated through validation in three independent Bca cohorts: GSE69795, GSE70691, and GSE31507.

### Model performance evaluation

We assessed the specificity and sensitivity of SIM by employing the R packages “Survival ROC” and “time ROC” to compute the area under the curve (AUC) using ROC curves. Additionally, the function of SIM was corroborated using two independent Bca validation cohorts, specifically GSE69795 and GSE31507.

### Gene expression plots and enrichment analysis

The R package “ggplot2” was utilized to generate volcano plots and heatmaps, which illustrate the distribution of DEGs and the expression levels of individual genes across each sample, respectively.

To identify DEGs, we utilized the entire genome as the background set and subsequently employed the R package “clusterProfiler” to complete Gene Ontology (GO) enrichment analysis (29). P-values below 0.05 are considered the threshold for statistical significance. In addition, the R package “ggplot2” (version 3.5.1) was employed to visualize the enrichment consequences.

To systematically investigate the gene functions, genomic information, and biochemical pathways of DEGs, we conducted a Kyoto Encyclopedia of Genes and Genomes (KEGG) enrichment analysis with the help of the R package “clusterProfiler”. Statistical significance of KEGG pathway was determined using a p-value cutoff of 0.05. Subsequently, the R package “ggplot2” (version 3.5.1) was employed to visualize the enrichment results.

To assess whether a predefined set of genes demonstrates steady variations between the two groups (high-SIMS and low-SIMS subgroups) and to identify instances where overarching trends are apparent despite the lack of significant changes in individual genes, the R package “clusterProfiler” alongside the GSEA software were utilized to carry out Gene Set Enrichment Analysis (GSEA) and enrichment analysis of DEGs between the two groups. The predefined gene set database utilized for this analysis was sourced from the MSigDB.

### Tumor microenvironment analysis

To study the differences in the TME across different groups, we employed the R package “xCell” to infer the proportions of various cell types within the TME based on gene expression data. This analysis encompassed differences in the proportions of stromal and immune cells. To ensure a precise assessment of immune cell infiltration ratios, we utilized eight distinct algorithms: ImmuCellAI, CIBERSORT, CIBERSORT.abs, quanTIseq, EPIC, MCPcounter, TIMER, and xCell. The results of ImmuCellAI were derived using the parameters of the single-sample gene set enrichment analysis (ssGSEA) method, while the other algorithms employed the respective R packages or online tools (http://timer.cistrome.org/). To assess the immune escape mechanisms within the TME and predict patients’ potential responses to immunotherapy through gene expression analysis, we utilized the online tool “Tumor Immune Dysfunction and Exclusion (TIDE)” to compute the immune response score for each patient. Additionally, we aimed to evaluate the prediction of immunotherapy response in high-SIMS and low-SIMS groups within this model. The accuracy of our model was determined by comparing the TIDE scores with the predicted immunotherapy responses.

### Gene mutation analysis

To investigate the genomic mutation distinctions between the two groups, we employed the R package “maftools” to explore and display the TCGA mutation annotation format (MAF) file (30). Additionally, we conducted a comprehensive suite of functional analyses, encompassing mutation frequency analysis, mutation type distribution assessment, and the calculation of tumor mutation burden (TMB).

### Drug sensitivity analysis

The Genomics of Drug Sensitivity in Cancer (GDSC) and the Cancer Therapeutics Response Portal (CTRP) are extensive publicly accessible databases designed to investigate the responsiveness of cancer cell lines to a diverse array of anticancer agents utilizing high-throughput screening methodologies. These databases provide substantial genomic and drug sensitivity datasets. In our research, we implemented the R package “oncoPredict” to conduct a comprehensive analysis of genomic data from TCGA, as well as from the GDSC and CTRP. Through this analysis, we were able to forecast the IC50 for each patient with different drugs. We conducted an analysis of drug resistance between the two patient cohorts by evaluating their differential sensitivity to various pharmacological agents.

## Results

### Identification of senescence-related differentially expressed genes of immunotherapy response sequencing in Bca

**Figure 1:**
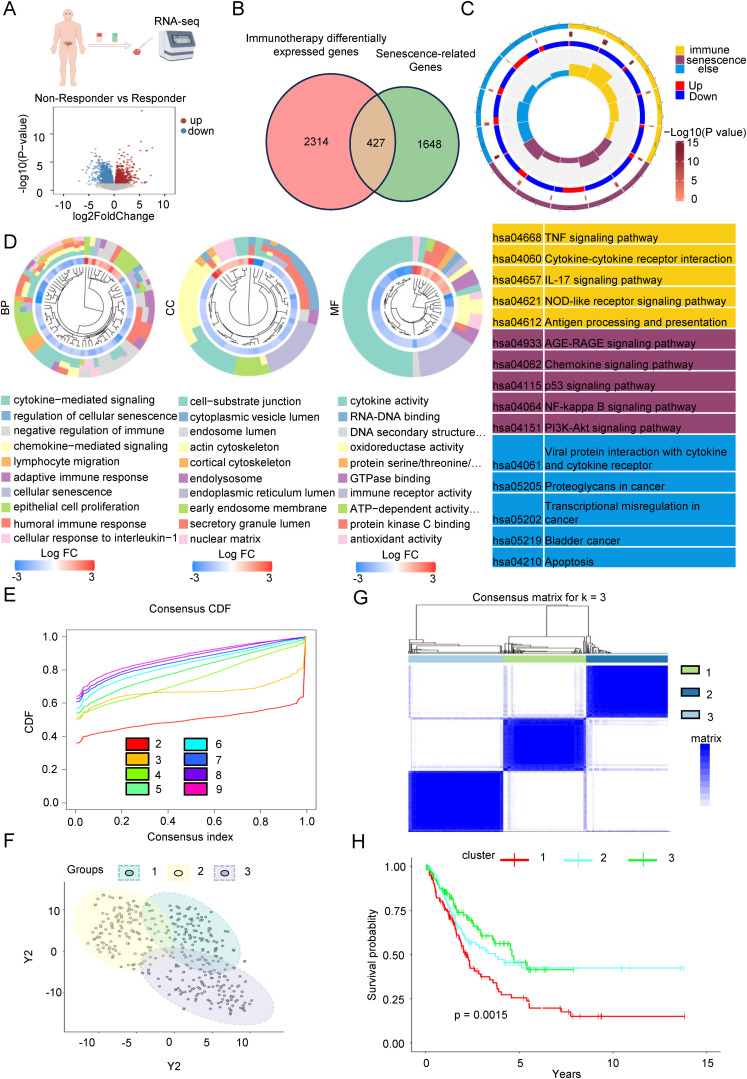
Identification of senescence-related differentially expressed genes of immunotherapy response sequencing in Bca. **(A)** we conducted transcriptome sequencing on cancerous tissues from Bca patients in both the immunotherapy response group and the non-response group who have been treated with low-dose paclitaxel in conjunction with immunotherapy in a real-world setting. The volcano plot shows the distribution of differentially expressed genes between the two groups. **(B)** The Venn diagram showed the intersection of genes related to differences in immune therapy response and aging-related differential genes, totaling 427 genes. **(C)** KEGG gene analysis showed that the intersecting genes are mainly enriched in cytokines, chemokines, cellular senescence, and immune response signaling pathways. **(D)** GO gene analysis showed that the intersecting genes are mainly enriched in pathways related to cellular senescence regulation, cytokine mediation and immune response signaling, cell adhesion, nuclear matrix, endoplasmic reticulum, cytokine activity, and RNA-DNA activity. **(E)** The figure showed the distribution of the consensus cumulative distribution function under different numbers of clustering clusters k. **(F)** Distribution of the three clusters after PCA. **(G)** Heatmap of the consistency matrix with k value (number of categories) equal to 3. **(H)** The KM survival curve analysis indicated that there are significant differences in the overall survival rates among the three groups of patients.

To investigate the underlying causes of variability in the effectiveness of immune checkpoint inhibitors, we conducted transcriptome sequencing on cancerous tissues from Bca patients in both the immunotherapy response group and the non-response group undergoing treatment with these inhibitors. The differential expression analysis, as illustrated in **Figure 1A**, identified 1,717 up-regulated and 1,024 down-regulated genes. Prior research has established a significant association between the TME and cellular senescence. Specifically, the microenvironment of pancreatic tumors has been shown to be characterized by inflammation induced by senescence (31). Consequently, we integrated the DEGs related to the immune response with the SRGs (**Supplementary Table 3**). As illustrated in the Venn diagram in **Figure 1B**, a total of 427 DEGs were recognized as being connected with senescence. Subsequently, we carried out an enrichment analysis on the expression of these SRDEGs using data from The TCGA database. The consequences of the KEGG enrichment analysis, depicted in **Figure 1C**, indicated that these SRDEGs were predominantly enriched in immune-related pathways, including the TNF signaling pathway, cytokine-cytokine receptor interaction, IL-17 signaling pathway, and antigen processing and presentation. Additionally, they were associated with senescence-related pathways, such as the AGE-RAGE signaling pathway and the p53 signaling pathway, as well as other pathways, including apoptosis. The consequences of the GO enrichment analysis presented in **Figure 1D** indicated that the Biological Process (BP) category is predominantly enriched in cytokine-mediated signaling, regulation of cellular senescence, and negative regulation of immunity. The Cellular Component (CC) category was primarily enriched in cell-substrate junctions, cytoplasmic vesicle lumens, and endosome lumens. Meanwhile, the Molecular Function (MF) category was chiefly enriched in cytokine activity, RNA-DNA binding, and immune receptor activity. To further investigate the intrinsic relationships among the significantly regulated differentially expressed genes (SRDEGs), we employed an unsupervised clustering method for classification (**Figure 1E**, **Supplementary Figures 1A, B**). According to the PAC algorithm, the best number of clusters (K) was verified to be 3. The clustering results, as depicted in **Figures 1F, G**, and **Supplementary Figures 1C, D**, demonstrated a robust clustering effect across the three categories. Based on gene expression profiles, patients were stratified into three distinct subgroups. Survival analysis (**Figure 1H**) revealed significant differences in overall survival among these subgroups, suggesting that gene expression variations are significantly connected to the prognosis of Bca patients.

To sum up, we discovered a senescence-related differential gene set by sequencing clinical samples undergoing immunotherapy and integrating this data with the SRGs. Subsequent analysis using the TCGA database revealed a noteworthy connection between these differential genes and the OS of patients.

### Construction of a senescence-related and immunotherapy related model to predict overall survival in Bca patients

**Figure 2:**
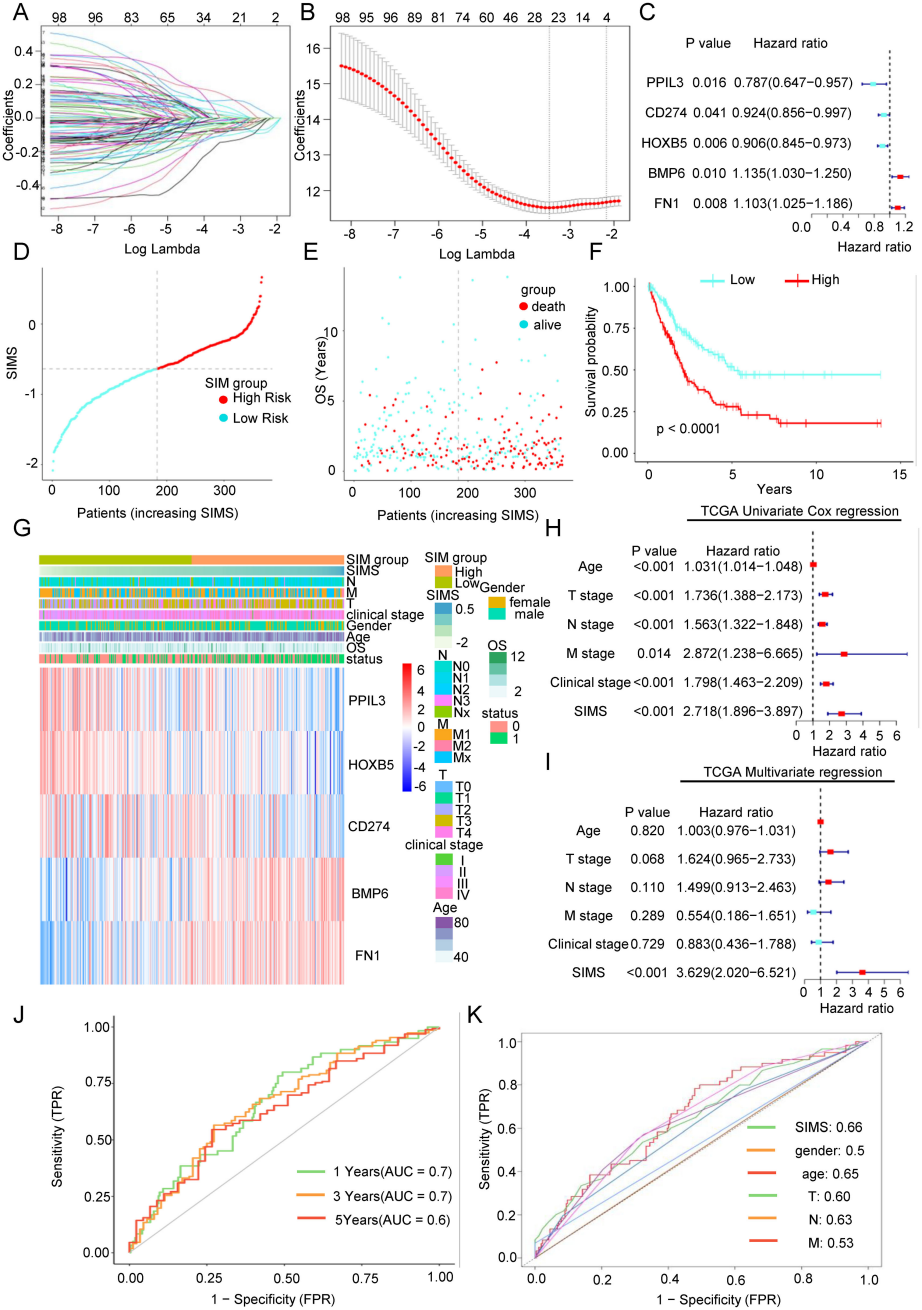
Construction of a senescence-related and immunotherapy related model to predict overall survival in Bca patients. **(A)** The regression coefficient path diagram presented in this article includes 101 variables, with each curve representing the trajectory of change for each independent variable’s coefficient. The vertical axis indicates the value of the coefficient, the lower horizontal axis represents log(λ), and the upper horizontal axis shows the number of non-zero coefficients in the model at that moment. **(B)** Cross-validation curve. The X-axis represents the logarithm of the penalty coefficient (log λ), while the Y-axis indicates the likelihood deviation. A smaller value on the Y-axis signifies a better fit of the equation. The number displayed at the top corresponds to the count of variables retained in the equation for various values of λ. **(C)** Forest plot was used to show the relationship between the five key genes obtained after multivariate Cox regression analysis and the overall survival rate of patients. **(D)** Scatter plot of the distribution of Bca patients as risk scores increase. **(F)** As the risk score increases, the scatter plot illustrating the distribution of Bca patients showed a clear division into high-risk and low-risk subgroups based on the median risk score. The low-risk subgroup was represented in turquoise, while the high-risk subgroup was indicated in red. **(E)** As the risk score increases, the scatter plot of the survival of Bca patients showed that green represents surviving patients and red represents dead patients. The lower left corner showed that the higher the risk score, the shorter the patient’s overall survival time and the higher the death rate. **(F)** The KM survival curve analysis indicated that there are significant differences in the overall survival rates between two subgroups. The high-risk subgroup exhibits significantly shorter survival time compared to the low-risk subgroup. **(G)** Distribution heat map of five key genes and clinical information in the model. **(H)** The univariate Cox regression analysis showed the relationship between the senescence-related and immunotherapy-related model scores (SIMS) and clinical factors and overall survival of patients. **(I)** The multivariate Cox regression analysis showed the relationship between SIMS and clinical factors and overall survival of patients. **(J)** The receiver operating characteristic (ROC) curve was used to describe the predictive value of the senescence-related and immunotherapy-related model (SIM) for patients at 1-, 3-, and 5-years. **(K)** The ROC curve was used to describe the predictive value of the SIM and other clinical factors for patients.

**Figure 3:**
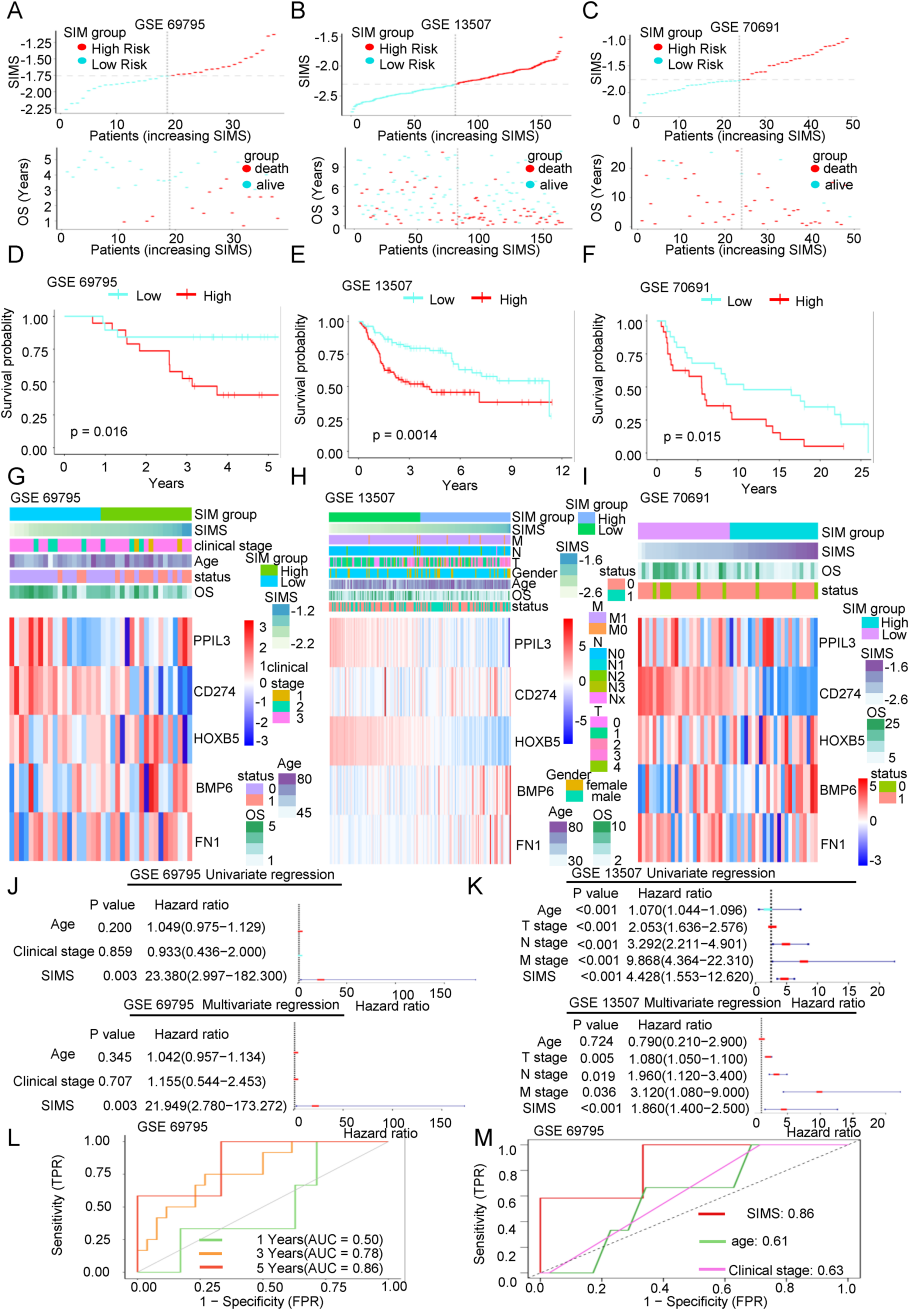
The accuracy of the SIM was verified by three independent databases. **(A-C)** Scatter plots of the distribution of risk scores and the relationship between risk scores and patient survival in three independent datasets, including GSE69795, GSE13507, and GSE70691. **(D-F)** KM survival curves were constructed to describe the relationship between risk scores and overall survival of patients in the three datasets. High-risk patients had a worse prognosis. **(G-I)** Heatmaps were used to show the distribution of genes in the SIM and clinical information in the three validation datasets. **(J, K)** Both the univariate and multivariate Cox regression analyses demonstrated the association between SIMS, clinical factors, and the overall survival of patients in the two validation datasets. **(L)** The ROC curve was used to describe the predictive value of the SIM for patients at 1-, 3-, and 5-years in GSE 69795 dataset. **(M)** The ROC curve was used to describe the predictive value of the SIM and other clinical factors for patients in GSE 69795 dataset.

To investigate which differentially expressed genes significantly influence patient prognosis and the efficacy of immunotherapy, we conducted a univariate regression analysis on 427 genes, identifying 101 genes associated with OS. Among these, 62 genes exhibited risk factors less than 1 (**Supplementary Figure 2**), indicating their protective nature, while the remaining 39 genes had risk factors greater than 1 (**Supplementary Figure 3A**), classifying them as risk factors. Subsequently, we employed LASSO regression analysis for bioinformatics refinement, reducing the gene set to 25, as depicted in **Figure 2B**. Ultimately, through multivariate regression analysis, we identified five key core genes: Bone Morphogenetic Protein 6 (BMP6), Fibronectin 1 (FN1), Programmed Death-Ligand 1 (CD274), Homeobox B5 (HOXB5), and Peptidylprolyl Isomerase Like 3 (PPIL3). The findings presented in **Figure 3B** illustrate the association between five key genes and Bca patient outcomes. Specifically, elevated expression levels of BMP6 and FN1 are linked to a poor prognosis, whereas higher expression levels of PPIL3, HOXB5, and CD274 correlate with a more favorable prognosis. Prognostic models were developed using multivariate regression analysis, focusing on senescence-related and immunotherapy-related model (SIM). The SIMS were calculated using the following formula: SIMS = expression of PPIL3 * (-0.13) + expression of CD274 * (-0.15) + expression of HOXB5 * (-0.10) + expression of BMP6 * 0.06 + expression of FN1 * 0.13. Bca were stratified into high-SIMS and low-SIMS subgroups based on the median SIMS score. **Figure 2D** depicts the distribution of patients across different risk score categories. Simultaneously, the high-SIMS subgroup predominantly occupied the region associated with shorter OS, whereas the low-SIMS subgroup was primarily located in the area corresponding to longer OS (**Figure 2E**). The KM survival analysis further elucidated the survival disparities between these two subgroups (**Figure 2F**). The findings corroborated previous observations, indicating that the high-SIMS subgroup experienced a much shorter OS compared to the low-SIMS subgroup.

We performed an in-depth study of the connection between the SIM and the clinical information of the patients. As depicted in **Figure 2G**, the expression of the five genes exhibited significant variability among different patients and was associated with the T stage, N stage, and M stage, indicating a notable relationship with the distribution of clinical stages. Through separate analyses of the relationship between the SIM and clinical data (**Supplementary Figure 1E**), we found, as anticipated, that the SIMS was significantly correlated with the T stage, N stage, M stage, clinical stage, and lymphovascular invasion. Furthermore, the consequences of the univariate regression analysis demonstrated that the SIM was statistically significant (**Figure 2H**). Simultaneously, the multivariate regression analysis results indicated that the model exhibited greater significance relative to other clinical factors (**Figure 2I**). To assess the effectiveness and accuracy of the SIM, we employed it to analyze the AUC for patients at 1-, 3-, and 5-years using data from the TCGA database (**Figure 2J**). Furthermore, a comparison with other clinical factors revealed that the model possesses strong predictive efficacy (**Figure 2K**).

In conclusion, we obtained a predictive SIM utilizing a differential gene set derived from real-world sequencing data, which demonstrates superior capability in forecasting overall survival compared to traditional clinical information.

### The accuracy of the SIM was verified by three independent databases

We have previously built the SIM using the TCGA database. Next, we aim to validate the diagnostic efficacy of the SIM using three independent datasets: GSE69795, GSE31507, and GSE70691. As illustrated in **Figures 3A–C**, we analyzed the distribution of patients based on increasing SIMS and categorized them into high-SIMS and low-SIMS groups in accordance with the median SIMS. Notably, in alignment with the consequences from the training set, patients in the high-SIMS subgroups across the three independent validation datasets predominantly exhibited shorter OS in comparison to those in the low-SIMS subgroups. To assess the statistical significance of these differences, we conducted Kaplan-Meier survival curve analyses for each dataset, as depicted in **Figures 3D–F**. The findings aligned with our initial hypotheses. In all three data sets, the high-SIMS subgroup had a worse survival prognosis than the low-SIMS subgroup, with statistically significant survival differences. Subsequently, as illustrated in **Figures 3G–I**, we presented the expression levels of the five genes included in the SIM using a heat map. The overall expression trends for these five genes were mostly aligned. Specifically, as the SIMS increased, the expression levels of BMP6 and FN1 presented a gradual rise, while the expression levels of PPIL3, CD274, and HOXB5 exhibited a gradual decline. Additionally, we provided an overview of the gene distribution within the SIM alongside the clinical information of the patients. To further elucidate the association between the SIM and other clinical variables, we conducted univariate regression analyses utilizing the SIM and the sole clinical information available in the GSE69795 and GSE13507 datasets. As depicted in **Figures 3J, K**, the SIM demonstrated robust predictive capability for overall survival (OS) in patients. Furthermore, the consequences of the multivariate regression analysis indicated that the SIM remained the most effective predictor when compared to other clinical factors in the GSE69795 and GSE13501 datasets. Subsequently, we calculated the 1-year, 3-year, and 5-year AUC values for SIM across the two datasets. As illustrated in **Figure 3L** and **Supplementary Figure 1F**, SIM demonstrates robust predictive capability, with the AUC in GSE69795 reaching 0.86. When compared to the AUCs of other clinical factors, the predictive performance of SIM remains superior (**Figure 3M**, **Supplementary Figure 1G**).

In conclusion, we have revalidated the predictive efficacy of SIM for OS in Bca using three independent databases, thereby minimizing the likelihood of random chance influencing the model construction process.

### Link between SIM and tumor microenvironment

**Figure 4:**
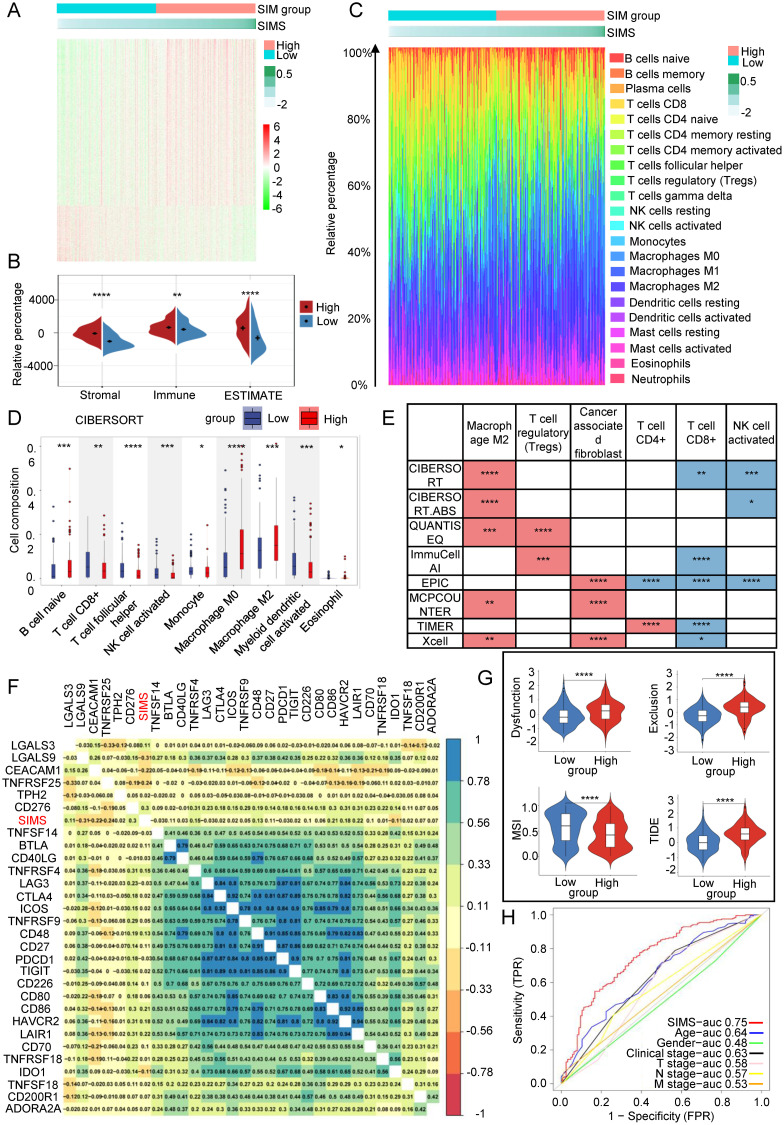
Link between SIM and tumor microenvironment. **(A)** Gene expression heatmaps were used to show the differential gene distribution between high-risk and low-risk subgroups of Bca patients. **(B)** The Estimation of STromal and Immune cells in MAlignant Tumor tissues using Expression data (ESTIMATE) algorithm was used to evaluate the purity of tumor samples. It is a method that uses gene expression features to infer the proportion of stroma and immune cells in tumor samples. **(C)** Percentage bar graphs were used to show the proportion of immune cells infiltrating in the tumor microenvironment for each patient. There was a significant difference in the infiltration of immune cells between the high-risk subgroup and the low-risk subgroup. **(D)** The CIBERSORT algorithm was used to calculate the infiltration ratio of immune cells in each patient, and the significant differences in immune cells between the two groups were shown in the figure. **(E)** Eight different algorithm results were used to present the differences between the two subgroups of immunosuppression-related cells and immune response-related cells. The red color in the figure represents the cell type with a higher infiltration ratio in the high-risk group than in the low-risk group. On the contrary, the blue color represents the cell type with a higher infiltration ratio in the low-risk group than in the high-risk group. **(F)** Correlation heatmaps were used to present the correlation between risk scores and immune checkpoint gene expression. **(G)** The Tumor Immune Dysfunction and Exclusion (TIDE) algorithm to evaluate immune infiltration within the tumor microenvironment and to predict the response to immunotherapy. We calculated the immune dysfunction score, immune exclusion score and microsatellite instability score for the two groups. **(H)** The ROC curve was used to describe the predictive value of the SIM for patients’ response to immunotherapy. (*p<0.05; **p<0.01; ***p<0.001;****p<0.0001).

The SIM model, developed based on immunotherapy response and SRGs, has been shown to influence the OS of Bca patients. To investigate the underlying mechanisms by which this model impacts patient OS, we conducted a differential genomic analysis of two distinct subgroups. The heat map illustrating the DEGs between these subgroups is presented in **Figure 4A**. We also assessed the proportion of immune cell infiltration in each patient, revealing noteworthy variances in immune cell infiltration within the TME between the subgroups, as depicted in **Figure 4B**. The GSEA of DEGs in immune-related pathways, as shown in **Supplementary Figures 3A, B**, indicated that the high-SIMS subgroup was predominantly enriched in gene sets associated with macrophages and helper T cells. In comparison, the low-SIMS subgroup was principally enriched in pathways related to immune response activation and specific Bca subgroups. The proportion of immune cell infiltration was measured for each patient, followed by a comparison of TME scores between the two subgroups. As illustrated in **Figure 4B**, the TME in the high-SIMS subgroup exhibited greater activity compared to the low-SIMS subgroup, characterized by elevated immune and stromal scores. **Figure 4C** highlights a significant disparity in immune cell infiltration within the TME between the subgroups. The data indicate that the high-SIMS subgroup has a higher prevalence of macrophages, helper T lymphocytes, and other cell infiltrations, whereas the low-risk subgroup predominantly features CD8+ lymphocyte infiltrations. To further elucidate and accurately compare the cellular differences in the tumor microenvironment of the two subgroups, we employed eight distinct algorithms to analyze the immune cell infiltration scores for each patient within each subgroup, as depicted in **Figures 4D, E**, and **Supplementary Figure 4**. Despite some variability among the algorithms, the overall trend remains consistent. The high-risk subgroup is characterized by the development of an immunosuppressive microenvironment that facilitates malignant tumor invasion, whereas the low-risk subgroup is associated with an immune infiltration microenvironment that inhibits tumor progression. Specifically, the presence of regulatory T (Treg) cells, M2 macrophages, and cancer-associated fibroblasts (CAFs) is significantly elevated in the TME of the high-SIMS subgroup in comparison to the low-SIMS subgroup. Conversely, the TME of the low-SIMS subgroup exhibits significantly higher levels of CD8+ lymphocytes, CD4+ lymphocytes, T follicular helper cells, and activated NK cells than that of the high-SIMS subgroup. Furthermore, we examined the relationship between SIMS and immune checkpoints. The gene expression heat map in **Figure 4F** reveals a strong correlation between SIMS and specific checkpoints, including LGALS9, TNFRSF25, CEACAM1, and CD86.

Subsequently, we employed the TIDE algorithm to evaluate immune infiltration within the tumor microenvironment and to predict the response to immunotherapy. As illustrated in **Figure 4G**, the high-SIMS subgroup exhibited elevated immune rejection and exhaustion scores in comparison to the low-SIMS subgroup. An elevated rejection score typically suggests that the tumor may obstruct T cell infiltration through various mechanisms, such as stromal fibrosis and the overexpression of angiogenic factors, potentially resulting in suboptimal immunotherapy outcomes. Similarly, a high exhaustion score indicates that, despite T cell infiltration, their functionality may be compromised by immunosuppressive signals, including the PD-1/PD-L1 and CTLA-4 pathways, thereby limiting the efficiency of immunotherapy. Notably, the low-SIMS subgroup demonstrated a higher Microsatellite Instability (MSI) score relative to the high-risk subgroup. A strong link often exists between high MSI levels and a greater tumor mutation burden (TMB), which can result in the production of additional neoantigens, ultimately improving the effectiveness of immune checkpoint inhibitors. High MSI scores may be associated with lower TIDE scores, lower T cell inactivation, and less T cell rejection, indicating a stronger immune response in the tumor microenvironment. Overall, lower TIDE scores in the low-SIMS subgroup suggest that they have a higher chance of benefiting from immunotherapy, whereas patients in the high-SIMS subgroup respond poorly to immunotherapy. Therefore, SIM is expected to become a predictive model for immunotherapy. In order to evaluate its predictive efficacy, as shown in **Figure 4H**, the AUC of SIMS is the largest compared with other clinical factors, indicating that SIM also has outstanding predictive ability in predicting the effect of immunotherapy in Bca patients.

In summary, the SIM are intricately associated with the tumor microenvironment, which subsequently influences the OS of patients. Furthermore, SIM serve as predictive indicators for the response of Bca patients to immunotherapy.

### The relationship between SIM and tumor gene mutation

**Figure 5:**
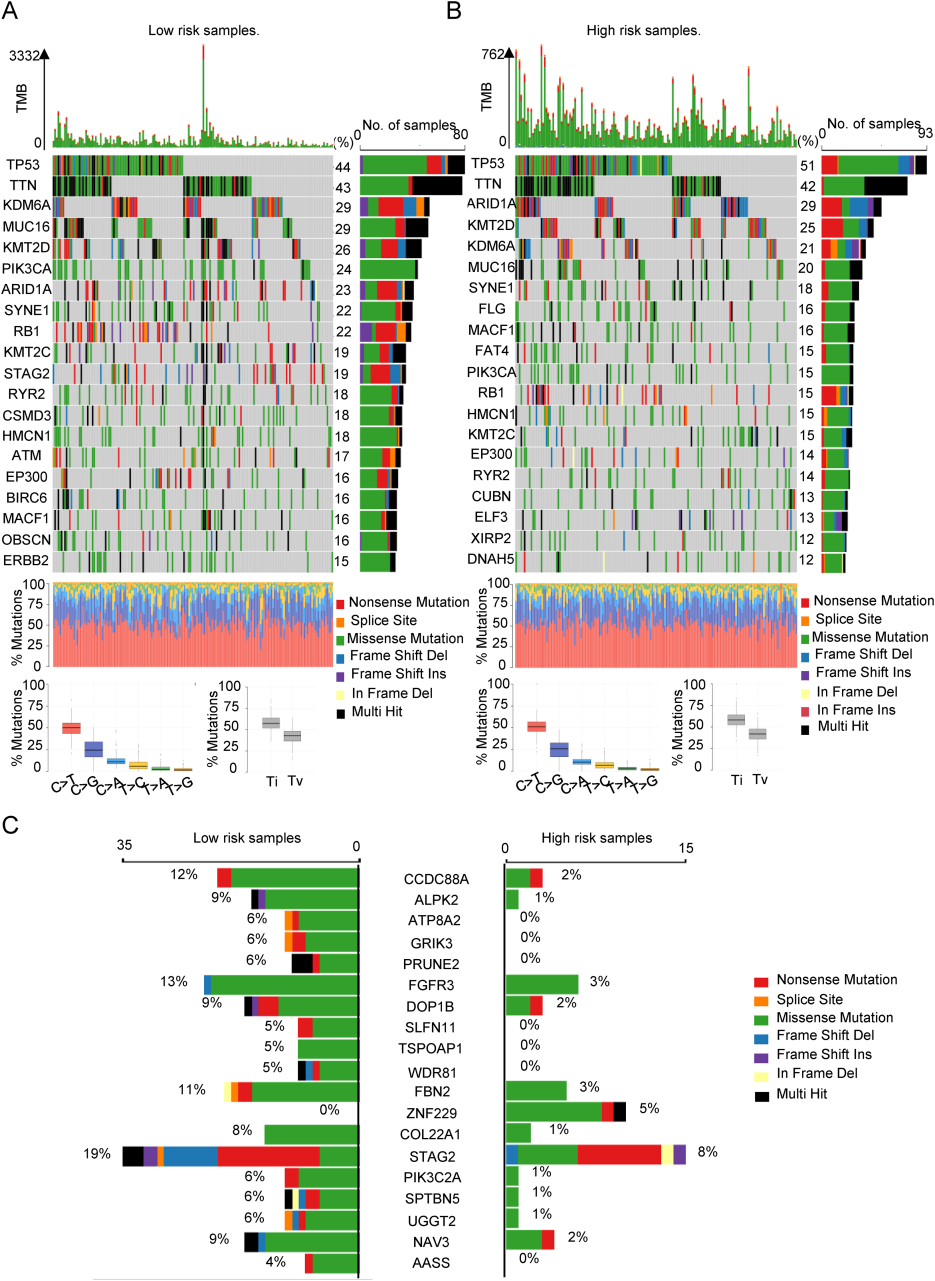
The relationship between SIM and tumor gene mutation. **(A)** The oncoplot was used to display the top 20 most frequently mutated genes in the low-risk subgroup of patients. **(B)** The oncoplot was used to display the top 20 most frequently mutated genes in the high-risk subgroup of patients. **(C)** Top 20 genes with the largest difference in mutation frequency between the two risk subgroups.

In our previous discussion, we noted that both the low-SIMS and high-SIMS subgroups exhibited elevated MSI scores. To further investigate the genetic mutation differences between these subgroups, we conducted an analysis using data from the TCGA database. As illustrated in **Figures 5A, B**, the mutation burden was significantly greater in the low-SIMS subgroup compared to the high-SIMS subgroup. By identifying the top 20 most frequently mutated genes within each subgroup, we observed substantial differences. Although TP53 and TTN were the most common mutations in both subgroups, subsequent genes varied considerably. Notably, the high-SIMS subgroup demonstrated a higher prevalence of ARID1A and KMT2D mutations, whereas the low-SIMS subgroup showed a greater incidence of KDM6A and MUC16 mutations. Additionally, we compared the top 20 genes exhibiting the largest mutation discrepancies between the two subgroups, as depicted in **Figure 5C**. These genes include CCDC88A, ALPK2, ATP8A2, and GRIK3. Such genetic mutations may play a pivotal role in influencing the TME, thereby impacting the OS of cancer patients.

In conclusion, we conducted an analysis of the distinct genomic mutations differentiating the two subgroups, which may significantly contribute to the progression and immunosuppression of Bca.

### SIM and drug response prediction

**Figure 6:**
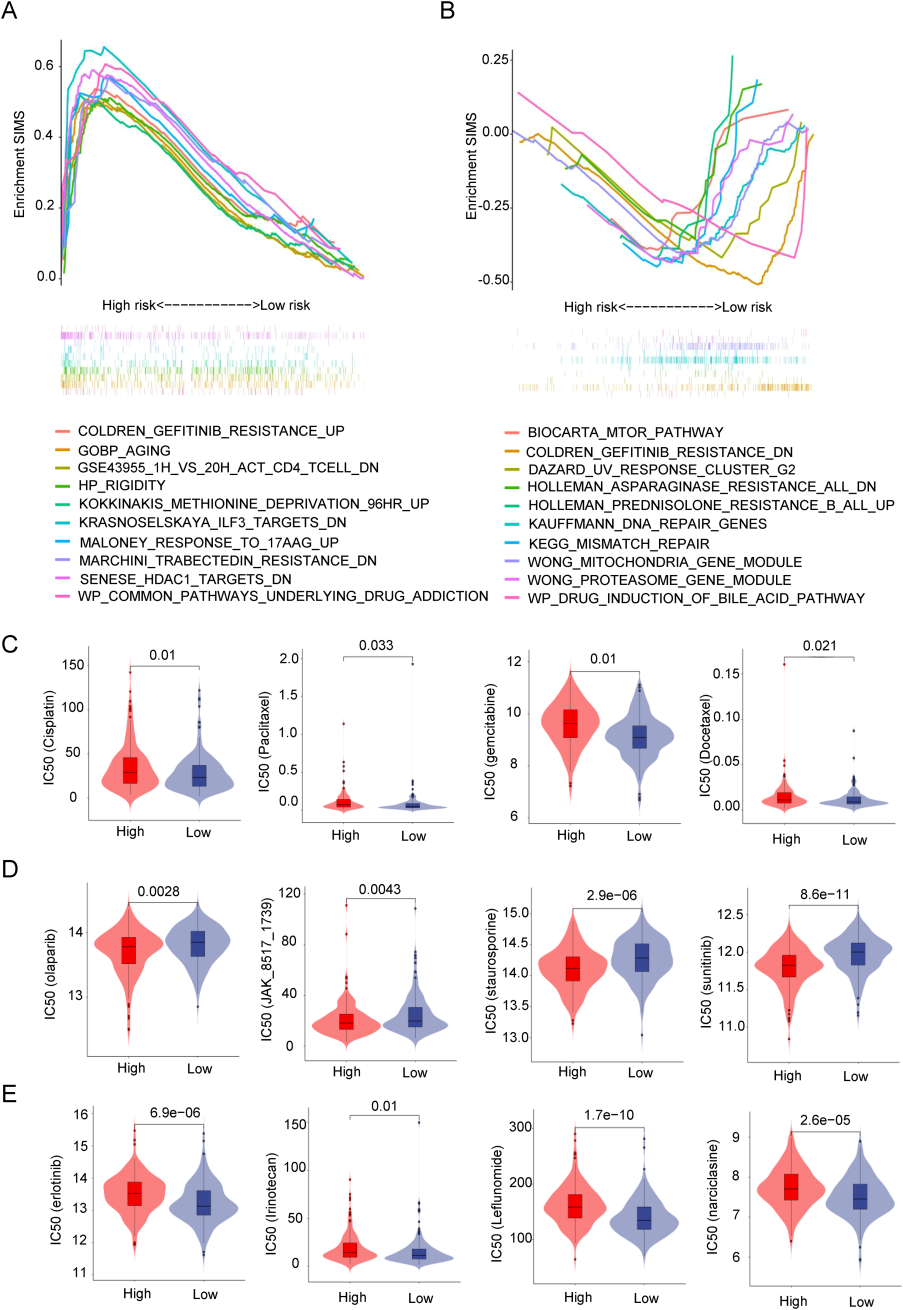
SIM and drug response prediction. **(A)** GSEA results were used to present drug-related signaling pathway gene sets enriched in high-risk subgroups. **(B)** GSEA results were used to present drug-related signaling pathway gene sets enriched in low-risk subgroups. **(C)** Analysis of differences in sensitivity between the two risk subgroups to commonly used chemotherapy drugs in clinical practice. **(D)** Prediction of small molecule drugs that are more sensitive in high-risk subgroups. **(E)** Prediction of small molecule drugs that are more sensitive in low-risk subgroups.

Given that SIM can predict immunotherapy response, it raises the question of whether SIM is also associated with responses to other drugs. As illustrated in **Figures 6A, B**, we employed GSEA to investigate the enrichment of drug-related pathways across different risk subgroups within SIM. The analysis revealed that the high-SIMS subgroup predominantly exhibited enrichment in pathways such as gefitinib resistance and aging, whereas the low-SIMS subgroup showed enrichment in pathways associated to DNA damage repair and drug response. Subsequently, we utilized R software and associated packages to assess the sensitivity of these subgroups to various drugs. As depicted in **Figure 6C**, individuals in the high-SIMS subgroup demonstrated insensitivity to several commonly used chemotherapeutic agents, including cisplatin, gemcitabine, paclitaxel, and docetaxel. Concurrently, we identified potential therapeutic agents for the high-SIMS subgroup. The results of **Figure 6D** indicated that this subgroup exhibited increased sensitivity to olaparib, JAK_8517_1739, staurosporine, and sunitinib. Conversely, as shown in **Figure 6E**, the low-SIMS subgroup was more responsive to erlotinib, irinotecan, leflunomide, and narciclasine.

Overall, by predicting drug sensitivity in the two subgroups, it was discovered that SIM influences not only the response to immunotherapy but also significantly affects the responses to other medications. By examining the drugs sensitive to different subgroups, more appropriate medications can be chosen for patients, allowing for a personalized treatment plan.

### Analyzing SIM in the real world

**Figure 7:**
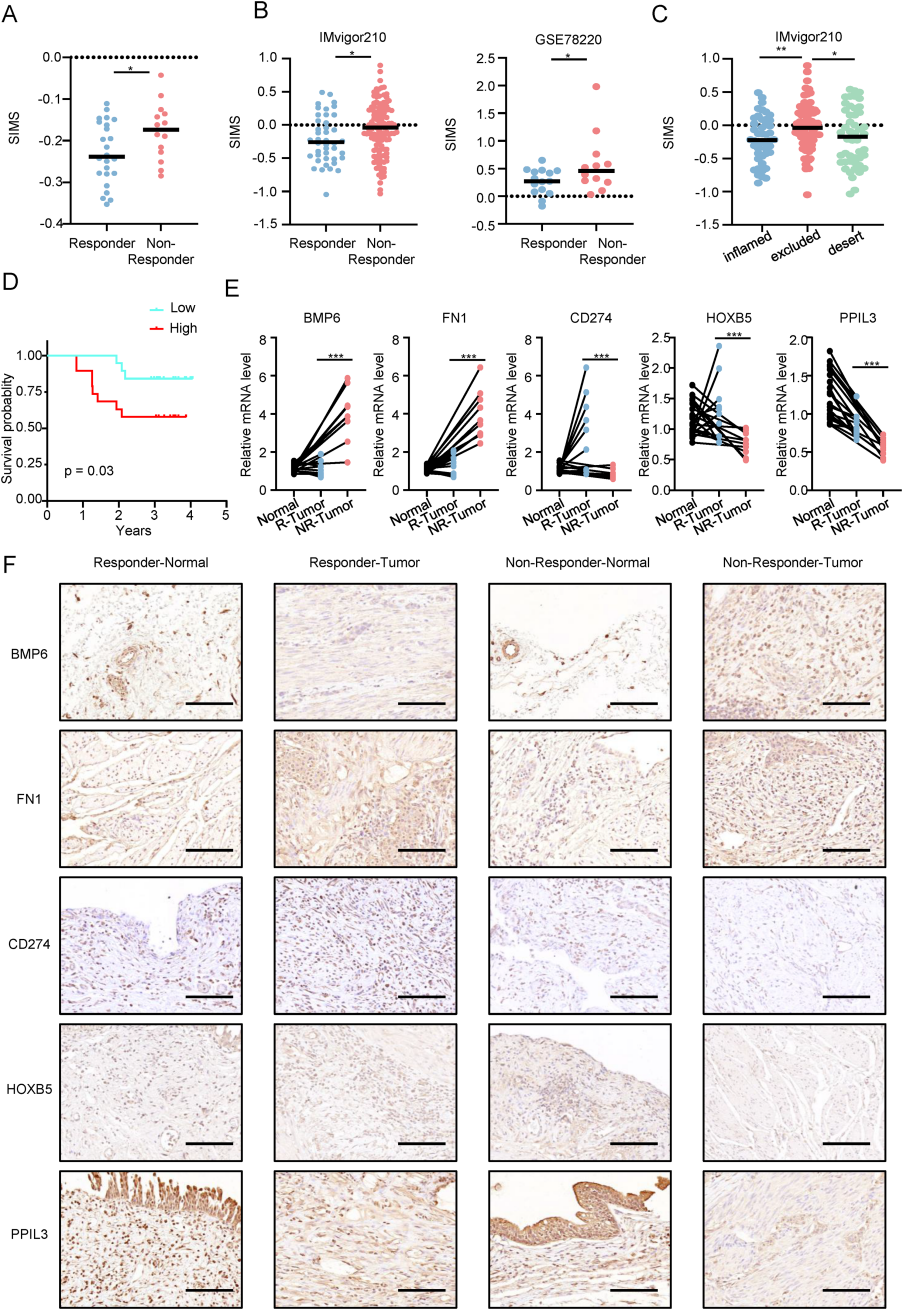
Analyzing SIM in the real world. **(A)** Differences in the senescence-related and immunotherapy-related model scores (SIMS) among patients with different immunotherapy responses in the real world. **(B)** Differences in the SIMS among patients with different immunotherapy responses in IMvigor210 and GSE78220. **(C)** Differences in risk scores among three types of patients in the IMvigor210 database. **(D)** In the real world, KM survival curves are used to confirm that patients in high-risk subgroups have lower overall survival and worse prognosis. **(E)** The results of PCR were used to present the expression of 5 genes in the model. **(F)** Immunohistochemistry results show the expression of 5 genes in the model in 10 patients (5 in the response group and 5 in the non-response group). The figure shows a representative image, and the scale bar is 100 μm. (*p<0.05; **p<0.01; ***p<0.001).

First, we verified the relationship between SIM and immune response in Real World. As shown in **Figure 7A**, the SIRS scores of patients in response group were higher than those of patients the non-response group. We have proved our point again (**Figure 7B**) in the well-known immune response database (IMvigor210) and GSE78220 database. At the same time, in **Figure 7C**, SIMS in patients with immune exclusion is the highest, which is consistent with our previous research results.

As shown in **Figure 7D**, in the real world, the KM survival curve is used to confirm that patients in the high-SIMS subgroup have lower overall survival and worse prognosis. Next, we explored the expression of five genes in SIM at the RNA level (**Figure 7E**). Patients who did not respond to immunotherapy had cancer tissues with elevated BMP6 and FN1 expression, in contrast to those who responded, where CD274, HOXB5, and PPIL3 were more expressed. Notably, PPIL3 expression was lower in cancer tissues for both patient groups. Finally, we studied the protein expression levels of five genes in SIM in Bca patients by immunohistochemistry (**Figure 7F**). We found that the protein expression levels for the five genes were generally consistent with their RNA expression levels.

To sum up, we confirmed SIM’s predictive capability for immunotherapy response in Bca patients using both real-world and online validation sets, and validated the multi-omic gene expression in SIM in real-world scenarios.

### PPIL3 inhibited proliferation and promoted senescence of Bca cells

**Figure 8:**
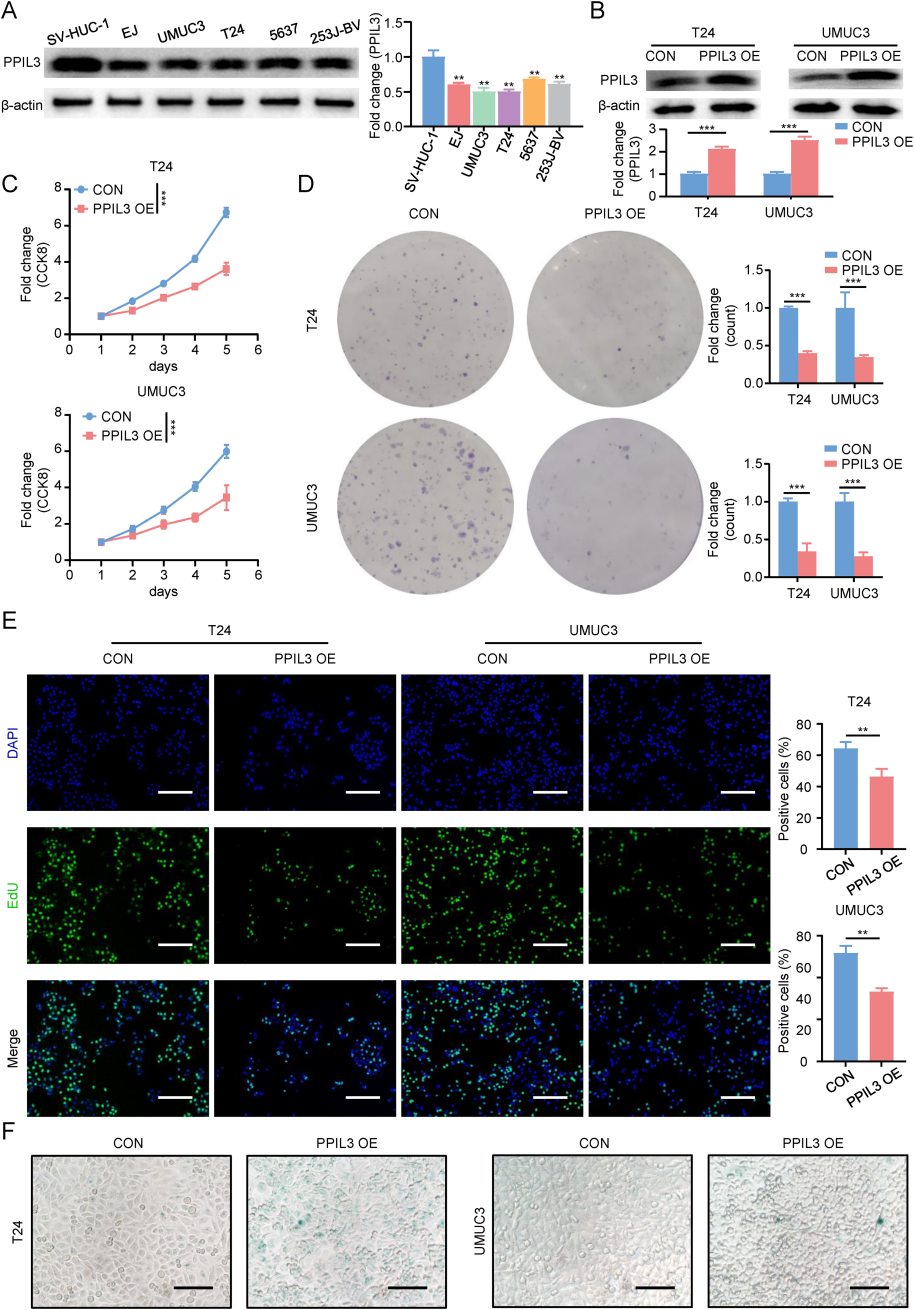
PPIL3 inhibited proliferation and promoted senescence of bladder cancer cells. **(A)**The results of western blot showed that PPIL3 expression was decreased in Bca cell lines. **(B)** The results of western blot showed that the PPIL3 stable overexpression cell lines we constructed was successfully constructed. **(C)** The results of CCK8 showed that the proliferation and survival ability of PPIL3 stably overexpressing cell lines was inhibited. **(D)** The results of plate cloning results showed that the clone formation ability of PPIL3 stably overexpressing cell lines was inhibited. **(E)** 5-Ethynyl-2’- deoxyuridine (EdU) assay for measuring the proliferation ability of T24 cells and UMUC3 cells PPIL3 overexpression or not. The cell proliferation ability of the PPIL3-overexpressing groups was significantly lower than the control groups. (Student’s t test; *p<0.05; **p<0.01; ***p<0.001.) The scale bar is 100 μm. **(F)** We measured the senescence marker β-galactosidase in T24 and UMUC3 cells after PPIL3 overexpression. The results indicated that elevated expression of PPIL3 facilitated the process of cellular senescence in Bca cells. The scale bar is 50 μm.

Given that previous studies have found that PPIL3 has the lowest risk ratio in the model and that its expression in tumor tissue is lower than that in adjacent normal tissue, both in the immunotherapy response group and the immunotherapy non-response group, we further explored the effect of its expression on Bca cell lines in *in vitro* experiments. We first investigated the expression of PPIL3 in Bca cell lines. Western blot results showed that PPIL3 was lowly expressed in Bca cells (**Figure 8A**). Next, as shown in **Figure 8B**, we constructed PPIL3 stably overexpressing Bca cell lines, T24 and UMUC3 cells. The CCK8 results in **Figure 8C** revealed that after PPIL3 overexpression, the proliferation and survival ability of Bca cells was significantly inhibited. Then we verified the important role of PPIL3 in inhibiting the clone-forming ability of Bca cells in a plate cloning experiment. The T24 and UMUC3 cells in the PPIL3 overexpression group were significantly inhibited (**Figure 8D**). We verified the inhibitory effect of PPIL3 on the proliferation of Bca cells again through EdU experiments (**Figure 8E**). Afterward, we measured the concentration of the senescence marker β-galactosidase and the expression of P16 and P21 in these two Bca cells. The results indicated that elevated expression of PPIL3 facilitated the process of cellular senescence in Bca cells (**Figure 8F**, **Supplementary Figure 5**).

In summary, we believed that PPIL3 inhibits Bca cell proliferation and promotes the formation of an anti-tumor immune microenvironment by inducing senescence.

## Discussion

Current clinical decision-making for Bca mainly relies on TNM staging and molecular classification, but lacks reliable markers for predicting immunotherapy response. Biomarkers such as PD-L1 expression and TMB recommended by the NCCN/EAU guidelines have high heterogeneity and limited predictive efficacy in clinical applications (32). The SIM constructed in this study integrates the dual characteristics of the aging microenvironment and immune response, and its AUC in independent cohorts is significantly better than traditional clinical parameters (**Figures 2K**, **3M**). More importantly, SIM can directly predict immunotherapy response (**Figures 4H**, **7A, B**), providing new guidance for the precise stratification of immunotherapy.

Cell senescence is a complex biological process, which is usually caused by stress, damage or proliferation stimulation of cells. In addition to their role in normal physiological processes, senescent cells greatly affect tumor growth and the immune microenvironment (21). Over the recent years, a rising amount of research has demonstrated that senescent cells play a dual role in the TME, which may inhibit the occurrence of tumors and facilitate the advancement of tumors (33–35). Therefore, exploring the intrinsic relationship between cell senescence and TME can provide a comprehensive solution for tumor treatment research.

Emerging drugs control cancer progression by enhancing oxidative stress-induced senescence (36). Albumin-paclitaxel has an important effect on cell cycle and cell senescence. At the same time, our previous studies have found that low-dose albumin-paclitaxel combined with immunotherapy has a good therapeutic effect in the treatment of Bca (37, 38), so our research subjects are Bca patients who choose a low-dose nab-paclitaxel combined with tislelizumab treatment regimen. Nonetheless, systematic and comprehensive studies on the inherent synergistic mechanism between chemotherapy and immunotherapy are still lacking. Chemotherapy or radiotherapy can induce cancer cell senescence. Low-dose chemotherapy leads to the senescence state of tumor cells, while high-dose chemotherapy induces cell apoptosis (39). We jointly analyzed senescence-related genes and genes obtained from immunotherapy response sequencing, and finally obtained the SRDEGs.

Next, we found five key genes for constructing the model through multivariate COX regression, which are BMP6, FN1, PPIL3, CD274 and HOXB5. BMP6 and FN1 are risk factors of the model, while PPIL3, CD274 and HOXB5 are protective factors of the model. The role of BMP6 is significant in a range of biological processes, including bone formation, cell differentiation, proliferation, and apoptosis. BMP6 is fundamental components of the senescent secretome, necessary for the paracrine induction of senescence and identified as dormancy drivers (40). Moreover, BMP6 has been implicated in the TME, particularly in melanoma. Research indicates that increased BMP6 expression in melanoma cells can modulate the tumor milieu by inhibiting dermal mast cell recruitment, which in turn affects tumor progression (41). FN1 is a glycoprotein widely present in the extracellular matrix and contributes significantly to biological processes such as cell adhesion, senescence (42), migration, proliferation and differentiation. Research indicates that FN1 significantly promotes the invasive and metastatic abilities of gastric cancer cells (43). PPIL3 is a protein belonging to the cyclophilin family, a class of enzymes that catalyze the cis-trans isomerization of proline residues in peptide chains and are involved in a variety of intracellular processes, including protein folding, signal transduction, and immune regulation (44). Studies have revealed that the expression of PPIL3 is significantly connected with OS of breast cancer (45). CD274, a protein important for immune regulation, is chiefly located on the surface of antigen-presenting cells, tumor cells, and different cell types. Although there are many studies on the role of CD274 in tumor immunity, for example, the expression of CD274 can inhibit anti-tumor immunity, especially by interacting with the PD-1 receptor on T cells, thereby reducing the activity and efficacy of T cells (46). Senescent cells tend to accumulate in the TME and evade immune surveillance by upregulating CD274, thereby promoting tumor progression (47). However, there are still new discoveries worth studying and thinking about. For example, CD274 can bind DNA, thereby controlling different pathways related to escaping immune surveillance or inflammation in the TME, playing a dual role (48). The transcription factor HOXB5, which belongs to the Homeobox gene family, has a significant role in the regulation of cell differentiation, tissue formation, and organ development. The expression of HOXB5 is closely associated with the metastasis of colorectal cancer (49), breast cancer (50), and hepatocellular carcinoma (51).

Next, we categorized the patients into a high-SIMS subgroup and a low-SIMS subgroup in accordance with the SIMS of the SIM, and further explored the intrinsic mechanism of the model in predicting Bca OS by using the DEGs between the two groups. Through bioinformatics analysis, we found that the immune infiltration score and stromal score of the TME in the high-SIMS group were notably higher than those in the low-SIMS group. To further explore the intrinsic cellular differences in the TME between the two groups of patients, we used 8 different algorithms to obtain the scores of each cell in each patient. We found that the infiltration ratios of CD4^+^T cells, CD8^+^T cells and activated NK cells in the TME of patients in the low SIMS group were large, while the infiltration ratios of M2 macrophages, CAFs and Tregs in the TME of patients in the high-SIMS group were large. On the one hand, CD4^+^ T cells (52), CD8^+^ T cells (53), and activated NK cells (54) play crucial roles in the TME, and they are essential for orchestrating immune responses and can promote immunity, influencing the efficiency of cancer immunotherapy. On the other hand, CAFs-derived IL-6 has been shown to cooperate with GM-CSF to induce M2-TAMs, which are associated with immunosuppressive functions (55). M2 macrophages, in turn, can further enhance the recruitment and activation of Tregs, creating a feedback loop that reinforces the immunosuppressive TME (56). In conclusion, we believe that patients in the high-SIMS group presented a more suppressive TME than those in the low-SIMS group, which was associated with their worse OS.

We continued to investigate the differences in response to immunotherapy between the two groups and found that patients in the high-SIMS subgroup had higher immune exclusion scores and immune dysfunction scores than those in the low-SIMS subgroup, but lower MSI scores. Consistent with our expectations, patients belonging to the low-SIMS subgroup had a higher likelihood of benefiting from immunotherapy. More importantly, compared with other information, our SIM served as the most effective independent predictor of patients’ responses to immunotherapy.

Given that the low-SIMS subgroup had a lower MSI score, we further explored the differences in genomic mutations between the two groups using bioinformatics algorithms. Recent studies have demonstrated that tumors exhibiting high levels of MSI often correlate with high TMB, which can enhance the likelihood of a favorable reaction to treatments aimed at the PD-1/PD-L1 pathway (57). High TMB is associated with a greater likelihood of generating neoantigens, which can boost the immune system’s power to identify and destroy tumor cells (58). Higher TMB in the low-SIMS group may be the key to better response to immunotherapy.

The genomic landscape characterized by clonal heterogeneity and the regulation of mRNA stability (59) are key drivers of the development of drug resistance in Bca (60). Xie R et al.’s study (61) revealed the mechanism of NAT10-mediated mRNA stabilization in Bca, laying the foundation for Nat10 as a therapeutic target to overcome cisplatin resistance in Bca. Considering that the high SIMS subgroup has lower sensitivity to first-line or commonly used chemotherapy drugs such as cisplatin, gemcitabine, and paclitaxel, we then looked for small molecule drugs to which patients in the high-SIMS subgroup exhibited increased sensitivity, including olaparib, JAK_8517_1739, staurosporine, and sunitinib. Olaparib is a poly (ADP-ribose) polymerase (PARP) inhibitor that has been used in the treatment of prostate cancer (62), breast cancer (63) and other cancers. JAK_8517_1739, staurosporine, and sunitinib are different kinase inhibitors, and these small molecule drugs are expected to become ideal treatment options for patients in the high-SIMS subgroup. In addition, we discovered that patients in the low-SIMS subgroup were sensitive to erlotinib, irinotecan, leflunomide, and narciclasine.

Finally, we validated the prediction of SIM for patients’ response to immunotherapy in real-world and online databases, and verified the expression of genes in SIM in different tissues of different Bca patients in the real world. At the same time, we explored the important role of PPIL3 in inducing senescence and inhibiting proliferation of Bca cells *in vitro*.

Of course, some limitations are also present in this study. This study is only a single-center study in the real world and lacks multi-center large-sample verification. Secondly, there is a lack of relevant cell experimental verification and more detailed mechanism research on the effect of gene expression in SIM on OS of Bca.

In general, our study has obtained a prediction model related to senescence and immunity to predict the OS and treatment response of Bca patients, which has important guiding significance for the exploration of treatment options. At the same time, our results revealed that PPIL3 is expected to become an important target for the treatment and prognosis of Bca.

## Data Availability

The raw data supporting the conclusions of this article will be made available by the authors, without undue reservation.

## Supplementary material

Supplementary Table 1 The information of primers and antibodies used in the experiments.

Supplementary Table 2 The information of differentially expressed genes (DEGs) from sequencing data analysis results.

Supplementary Table 3 The information of senescence-related datasets.

Supplementary Figure 1
**(A)** The Delta area chart was used to explore the relative change in the area under the cumulative distribution function curve when comparing k and k-1. **(B)** The Tracking Plot was utilized to examine the variations in the sample class across different values of k. In this plot, the columns represented the samples, the rows indicated each k value, and the colors denoted the categories within the consistency matrix. **(C)** Heatmap of the consistency matrix with k value (number of categories) equal to 4. **(D)** The Two-dimensional scatter plot was used to explore the distribution differences among three groups of patients. **(E)** Distribution of correlation between senescence-related and immunotherapy-related model scores (SIMS) and patients' clinical information. The SIMS was significantly correlated with the T stage, N stage, M stage, clinical stage, and lymphovascular invasion. **(F)** The ROC curve was used to describe the predictive value of the SIM for patients at 1-, 3-, and 5-years in GSE 13507 dataset. **(G)** The ROC curve was used to describe the predictive value of the SIM and other clinical factors for patients in GSE 13507 dataset.

Supplementary Figure 2 Univariate Cox regression analysis was used to construct senescence-related and immunotherapy-related models to predict the overall survival of Bca patients. The Forest plots were used to show protective genes among the differentially responsive genes to immunotherapy associated with aging, which were positively correlated with the overall survival of patients.

Supplementary Figure 3 Construction and mechanism exploration of senescence-related and immunotherapy response-related models. **(A)** The Forest plots were used to show risk genes among the differentially responsive genes to immunotherapy associated with aging, which were negatively correlated with the overall survival of patients. **(B)** GSEA results were used to present immune-related signaling pathway gene sets enriched in high-risk subgroups. **(C)** GSEA results were used to present immune-related signaling pathway gene sets enriched in low-risk subgroups.

Supplementary Figure 4 Different algorithms were used to calculate the proportion of immune cell infiltration in Bca patients. **(A)** QUANTISEQ algorithm. **(B)** ImmuCell AI algorithm. **(C)** CIBERSORT.ABS algorithm. **(D)** EPIC algorithm. **(E)** MCPCOUNTER algorithm. **(F)** TIMER algorithm. **(F)** Xcell algorithm.

Supplementary Figure 5 The results of western blot showed that the expression of P16 and P21 in the PPIL3 stable overexpression cell lines.
