# Supplementary figures and images for: Senescence-associated signature based on immunotherapy response sequencing reveals PPIL3 as target for bladder cancer treatment and prognosis prediction
Source: Front Immunol. 2025 Jun 26;16:1613056. doi: 10.3389/fimmu.2025.1613056 (PMC12240782; doi:10.3389/fimmu.2025.1613056)

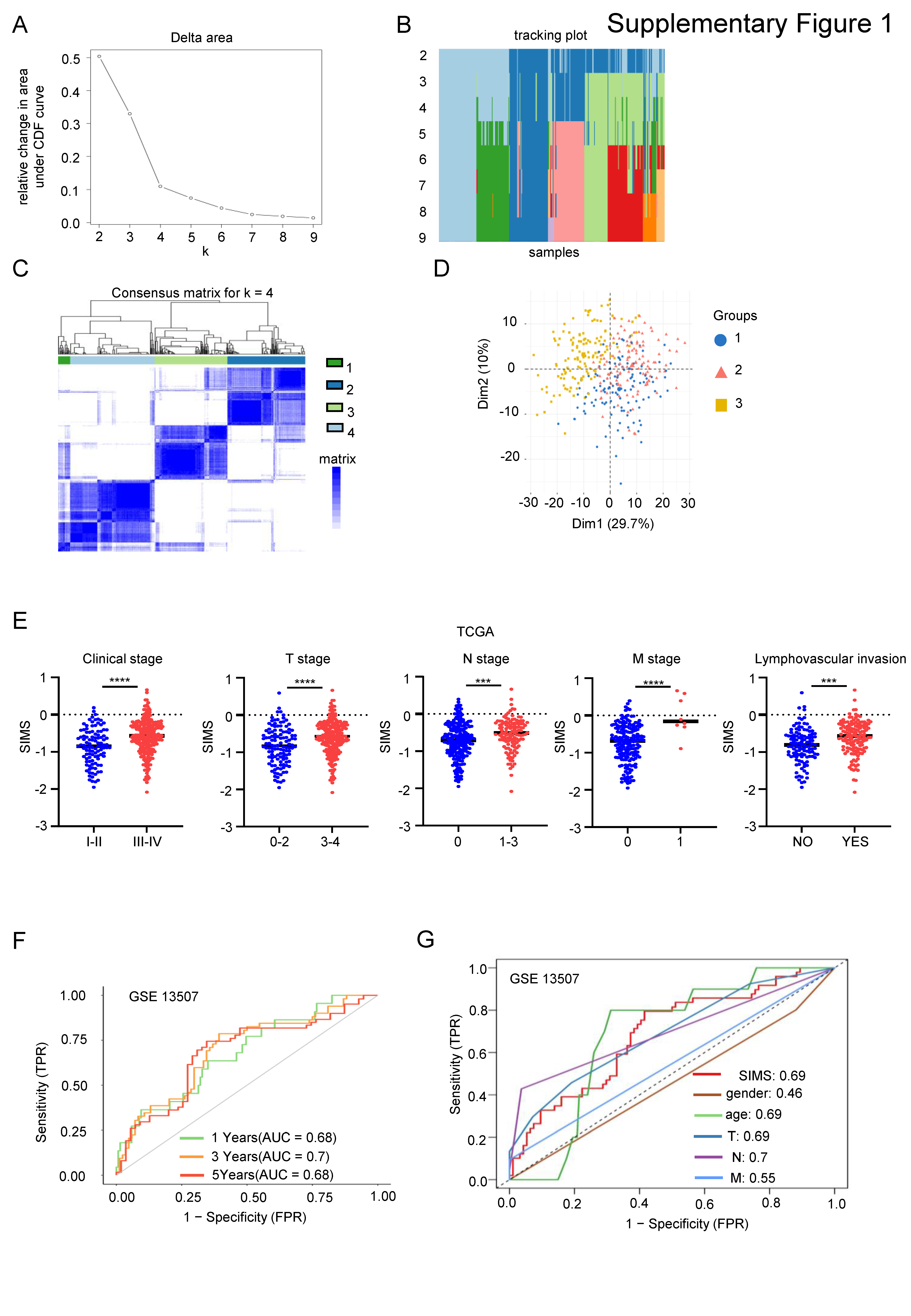

Supplement: Supplementary Figure 1 — (A) The Delta area chart was used to explore the relative change in the area under the cumulative distribution function curve when comparing k and k-1. (B) The Tracking Plot was utilized to examine the variations in the sample class across different values of k. In this plot, the columns represented the samples, the rows indicated each k value, and the colors denoted the categories within the consistency matrix. (C) Heatmap of the consistency matrix with k value (number of categories) equal to 4. (D) The Two-dimensional scatter plot was used to explore the distribution differences among three groups of patients. (E) Distribution of correlation between senescence-related and immunotherapy-related model scores (SIMS) and patients' clinical information. The SIMS was significantly correlated with the T stage, N stage, M stage, clinical stage, and lymphovascular invasion. (F) The ROC curve was used to describe the predictive value of the SIM for patients at 1-, 3-, and 5-years in GSE 13507 dataset. (G) The ROC curve was used to describe the predictive value of the SIM and other clinical factors for patients in GSE 13507 dataset. [file Image1.tif]

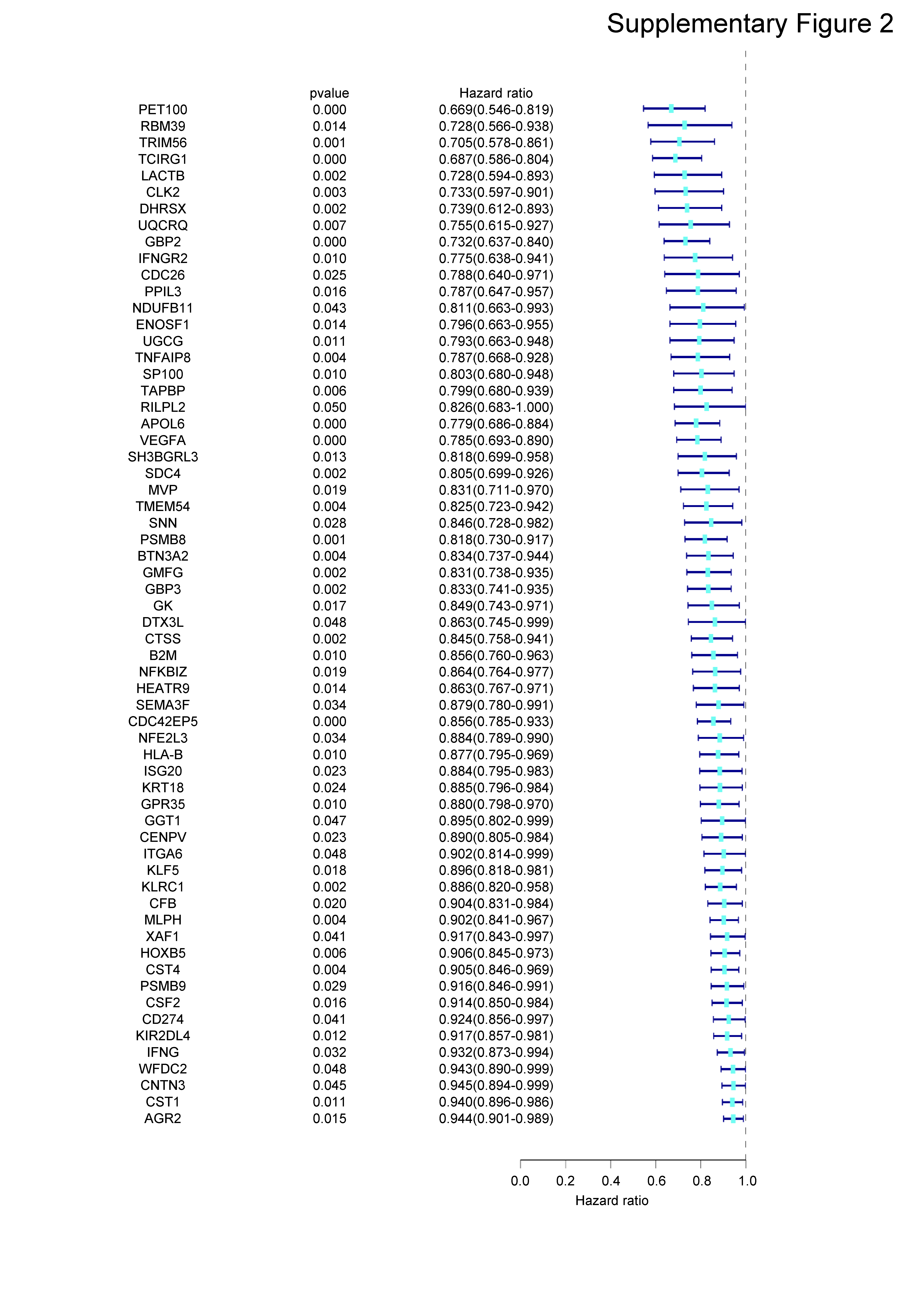

Supplement: Supplementary Figure 2 — Univariate Cox regression analysis was used to construct senescence-related and immunotherapy-related models to predict the overall survival of Bca patients. The Forest plots were used to show protective genes among the differentially responsive genes to immunotherapy associated with aging, which were positively correlated with the overall survival of patients. [file Image2.tif]

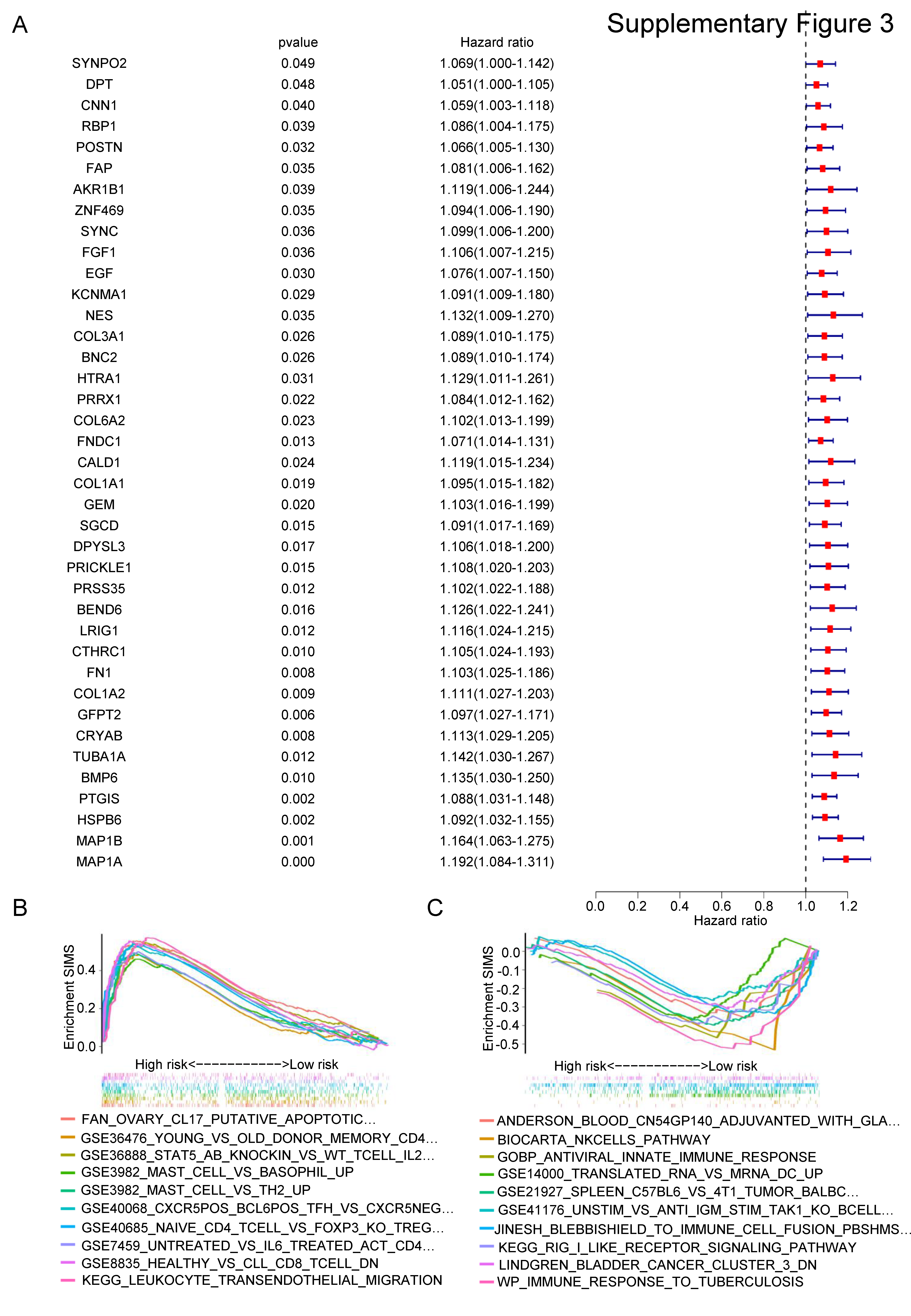

Supplement: Supplementary Figure 3 — Construction and mechanism exploration of senescence-related and immunotherapy response-related models. (A) The Forest plots were used to show risk genes among the differentially responsive genes to immunotherapy associated with aging, which were negatively correlated with the overall survival of patients. (B) GSEA results were used to present immune-related signaling pathway gene sets enriched in high-risk subgroups. (C) GSEA results were used to present immune-related signaling pathway gene sets enriched in low-risk subgroups. [file Image3.tif]

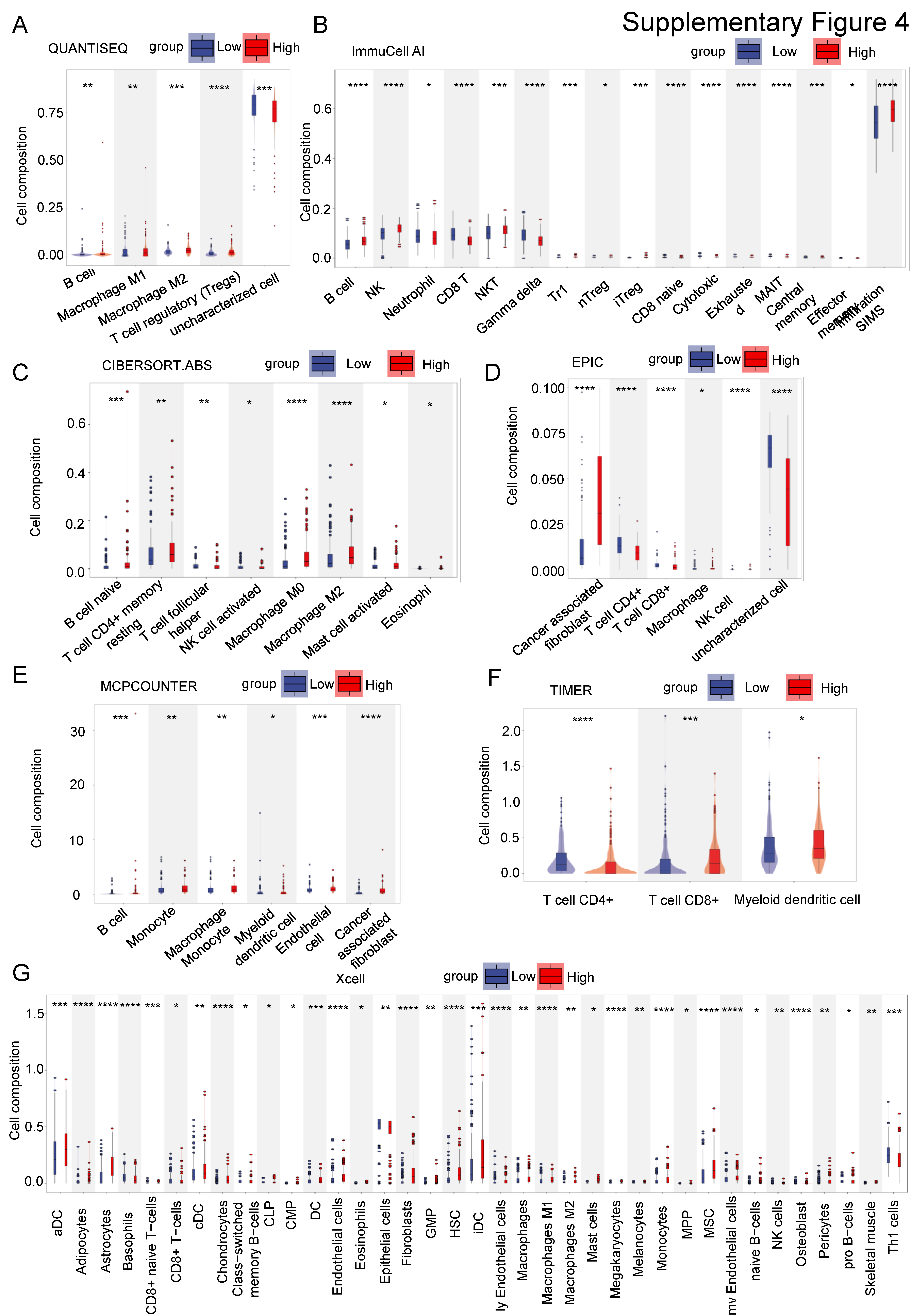

Supplement: Supplementary Figure 4 — Different algorithms were used to calculate the proportion of immune cell infiltration in Bca patients. (A) QUANTISEQ algorithm. (B) ImmuCell AI algorithm. (C) CIBERSORT.ABS algorithm. (D) EPIC algorithm. (E) MCPCOUNTER algorithm. (F) TIMER algorithm. (F) Xcell algorithm. [file Image4.tif]

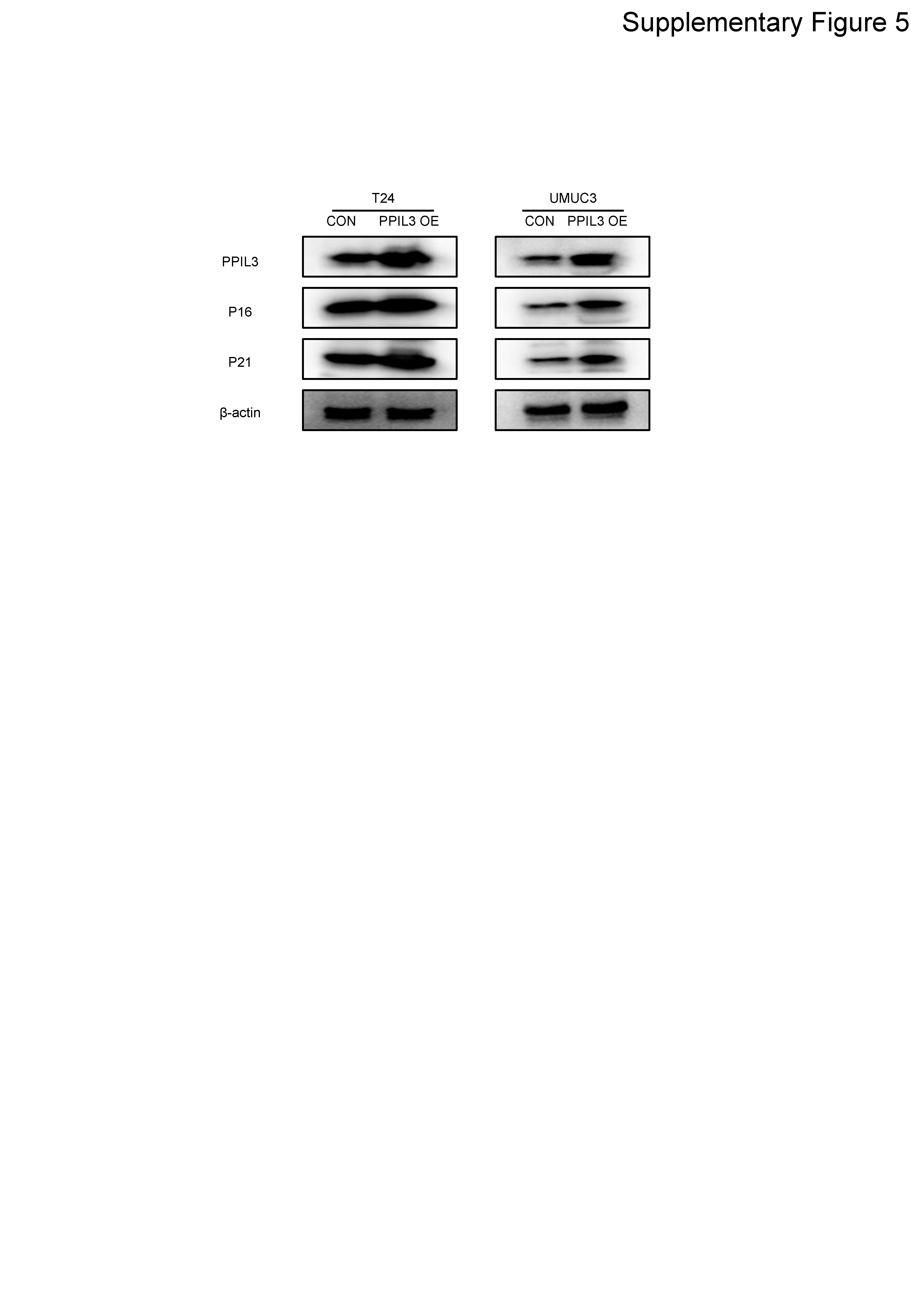

Supplement: Supplementary Figure 5 — The results of western blot showed that the expression of P16 and P21 in the PPIL3 stable overexpression cell lines. [file Image5.tiff]
